# Association between optic disc pallor and lacunar stroke

**DOI:** 10.1136/bmjno-2024-000789

**Published:** 2024-08-30

**Authors:** Samuel Gibbon, Fergus Doubal, Francesca Chappell, Joanna M Wardlaw, Baljean Dhillon, Thomas MacGillivray

**Affiliations:** 1 Centre for Clinical Brain Sciences, The University of Edinburgh, Edinburgh, UK; 2 Robert O Curle Ophthalmology Suite, Institute for Regeneration and Repair, The University of Edinburgh, Edinburgh, UK; 3 UK Dementia Research Institute Centre, The University of Edinburgh, Edinburgh, UK

**Keywords:** STROKE, NEURORADIOLOGY, NEUROOPHTHALMOLOGY

## Abstract

**Objective:**

To test for associations between optic disc pallor and two clinical variables: ischaemic stroke subtype (cortical and lacunar) and cerebral small vessel disease (SVD) scores in a cohort of hospital patients admitted with mild stroke (Mild Stroke Study 1).

**Methods:**

We used previously validated software, *PallorMetrics*, to quantify optic disc pallor in colour fundus photographs of patients diagnosed as having either cortical (n=92) or lacunar (n=92) stroke. We used logistic regression to assess the relationship between stroke type and disc pallor in several zones and ordinal logistic regression to assess the relationship between disc pallor and total SVD score. The left and right eyes were analysed separately.

**Results:**

In the right eye, independent of age, sex, disc area, hypertension and diabetes, increased optic disc pallor was significantly associated with lacunar stroke in all zones (for global pallor: OR per SD increase=1.55, 95% CI 1.11 to 2.17, p=0.011) and total SVD score in the temporal superior (standardised β=0.36, SE=0.15, p=0.020) and nasal-inferior zones (standardised β=0.44, SE=0.15, p=0.004) in the right eye. Weaker trends were observed in the left eye; however, these did not reach statistical significance.

**Conclusion:**

Optic disc pallor may be associated with SVD severity and lacunar stroke, which may reflect vascular damage to the optic nerve or its pathways. Our findings underscore the utility of colour fundus photography to learn more about SVD pathology.

**WHAT IS ALREADY KNOWN ON THIS TOPIC**

- Cerebral small vessel disease (SVD) causes lacunar stroke, which accounts for around 25% of all ischaemic strokes and differs from other ischaemic strokes in terms of prognosis, therapy and prevention strategies. The retina, particularly the optic disc, due to its vascular similarity to the brain, has been identified as a potential site for biomarkers that could help in understanding and diagnosing SVD and stroke.

**WHAT THIS STUDY ADDS**

- This study found that optic disc pallor is significantly associated with the lacunar stroke subtype (compared with atheromatous large artery cortical strokes) and higher SVD scores. This finding suggests that optic disc pallor could be a marker of chronic small vessel vascular impairment affecting the optic nerve and may serve as a non-invasive indicator of SVD severity.

**HOW THIS STUDY MIGHT AFFECT RESEARCH, PRACTICE OR POLICY**

- The association between optic disc pallor and SVD highlights another feature of retinal fundus imaging that can act as a convenient and non-invasive method for assessing brain health and SVD severity. This could potentially lead to improved diagnostic tools and more targeted therapeutic strategies to prevent SVD-related stroke and cognitive decline, influencing clinical practice and policy in stroke management and prevention.

## Introduction

 Cerebral small vessel disease (SVD) is an age-associated condition impacting the perforating small blood vessels of the central nervous system and constitutes a significant factor in the development of stroke, dementia and cognitive decline.^1^,^3^ In particular, SVD is a major cause of lacunar stroke,^4 5^ which accounts for around 25% of all ischaemic strokes^6 7^ and differs from other ischaemic strokes with regard to prognosis, therapy and prevention strategies.^68^,^10^ SVD can be detected in the brain with MRI. Key visible indicators of SVD include lacunes, microbleeds, white matter hyperintensities (WMHs) and enlarged perivascular spaces.^1^,^4^ However, MRI is expensive, with limited availability. Instead, the retina, owing to its homology with the brain,^11^ particularly with regard to the microvasculature, has become a target for biomarker discovery and mechanistic insight into SVD and stroke.^12^

Previous work using colour fundus photographs investigating the eye–brain connection in SVD and lacunar stroke has shown increased microvasculature signs in lacunar compared with cortical stroke, including wider retinal venules,^13 14^ decreased fractal dimension,^15^ narrower arterioles and greater arteriovenous nipping,^14^ highlighting the importance of vascular changes in lacunar stroke. Most other work looking at retinal features and stroke has also focused on the retinal vasculature.^13^,^18^ However, an under-researched feature of the retina that could provide further insight into SVD is the optic disc.

A pale disc, for instance, indicates optic atrophy and represents loss or damage to retinal ganglion cell (RGC) axons along the anterior visual pathway, and to some extent, a loss of vasculature.^19^ The quantity of RGC axons is captured by the retinal nerve fibre layer (RNFL), which can be measured with three-dimensional scanning technology, optical coherence tomography (OCT). Thinning of the peripapillary RNFL (pRNFL; circular OCT scan around the optic disc) has been associated with SVD,^12^ Alzheimer’s disease, mild cognitive impairment,^20^ increased risk of dementia,^21^ future cognitive decline^22^ and increased cardiovascular risk.^23^ One study looked directly at pRNFL defects and stroke,^24^ finding increased defects (and hence thinner pRNFL) in acute stroke compared with controls. However, research investigating optic disc pallor/atrophy and stroke is scant; we found just one study,^25^ with the authors reporting a high prevalence of optic atrophy in patients who had ischaemic stroke; stroke subtypes were not assessed.

One potential reason for the scarcity of research in this area could be the need for specialist ophthalmological assessment to diagnose optic atrophy/pallor, which can vary among observers.^26^ Recently, we developed an automatic method of obtaining continuous measurements of optic disc pallor from colour fundus photographs,^27^ which may act as a proxy for pRNFL thickness and the detection of defects. In the current study, we explore associations between optic disc pallor and two clinical features: ischaemic stroke subtype (cortical and lacunar) and total SVD scores in a prospective study of minor stroke and SVD.

## Methods

### Participants and image capture

The Mild Stroke Study 1 population has been described in detail elsewhere.^13 15 28^ Briefly, 220 patients who had experienced a clinical ischaemic or minor cortical stroke were recruited from a hospital stroke service in Edinburgh, UK. Due to legacy problems with data linkage, retinal fundus photographs were available for 186 patients. All patients underwent MRI (1.5 T; Signa LX; General Electric) at presentation and had retinal fundus images (Canon CR-DGi; Canon USA) taken within 4 weeks of stroke onset. The MRI sequence included axial diffusion-weighted, T2-weighted, fluid-attenuated inversion recovery (FLAIR) and gradient echo, while the retinal photography was six-field (including optic disc centred) of both eyes. Retinal imaging was carried out according to the Early Treatment Diabetic Retinopathy Study protocol,^29^ which always captured the right eye first, and administered 1% tropicamide as necessary (two drops per eye) to dilate the pupil. Prior to dilation, patients were screened for glaucoma and a history of glaucoma.

### Stroke classification

#### Clinical

A stroke physician (author FD) examined all patients and assessed stroke severity using the NIH Stroke Scale.^30^ The stroke clinical syndrome was classified as either lacunar or cortical based on the Oxfordshire Community Stroke Project classification.^31^ Briefly, cortical stroke was defined as a partial anterior circulation stroke syndrome or, if in the posterior circulation, visual field loss. Lacunar stroke was defined by pure motor weakness and/or sensory loss in the face and arm, arm and leg, or all three, as well as ataxic hemiparesis or clumsy hand dysarthria syndrome.^13 32^

#### Radiological

A consultant neuroradiologist (author JW) identified and located acute ischaemic stroke lesions (low signal on apparent diffusion coefficient (ADC) map, high signal on diffusion imaging), old ischaemic stroke lesions (high/low FLAIR signal, high T2 signal, no signal on diffusion imaging/ADC map) and old haemorrhages (low gradient-echo signal). Lacunar infarcts were defined as acute ischaemic lesions, round and under 2 cm in the centrum semiovale, internal capsule, brainstem or basal ganglia. Cortical lesions were defined as acute ischaemic lesions affecting cortical and adjacent subcortical tissue in a known cortical arterial branch territory, including striatocapsular infarcts (>2 cm diameter in the basal ganglia).^32^ Clinical ratings were combined with MRI-based measures (whether the recent infarct was cortical or lacunar) to produce a final stroke subtype classification. Where the two differed, the radiological classification was preferred.

### Total SVD score

Total SVD score (ordinal score of 0–4) was calculated according to Staal’s (2014) method,^33^ whereby one point was allocated for each of four MRI features of SVD: presence of lacunes, presence of microbleeds, moderate to severe enlarged perivascular spaces (grades 2–4) and WMHs (Fazekas score 2 or 3 for deep WMH and Fazekas score 3 for WMH extending into deep white matter).

### Optic disc pallor quantification

**Figure 1:**
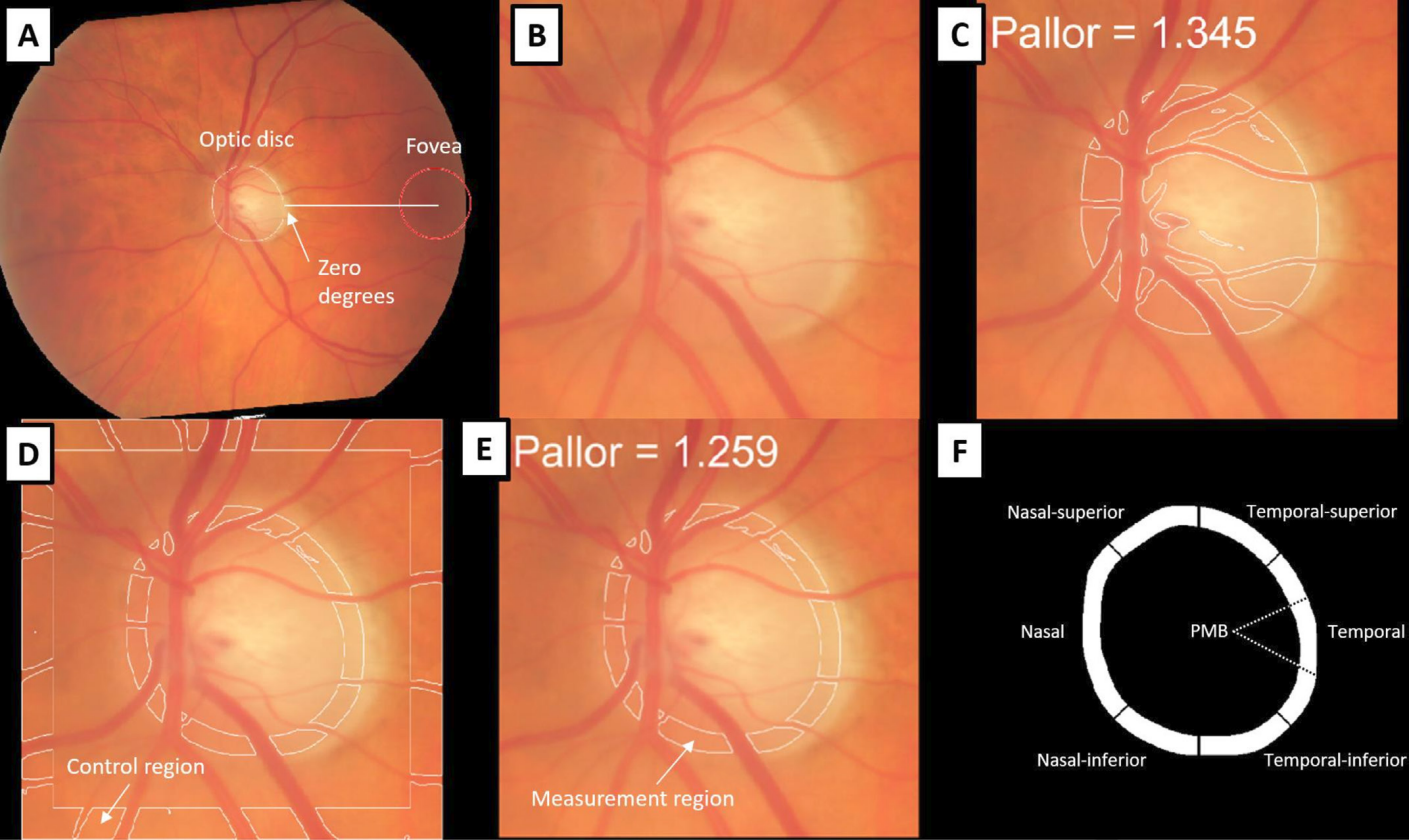
Analysing optic disc pallor in a fundus image. (**A**) Full image rotated along the optic disc-fovea line (fovea location is predicted to lie within the red circle). (**B**) Image cropped to the optic disc. (**C**) Segmented whole disc with measured pallor value. (**D**) Segmented measurement and control region. (**E**) Measurement region with global pallor value. (**F**) Diagram of zones. Note: Retinal vessels are excluded in (C, D and E). PMB, papillomacular bundle.

We generated continuous measures of optic disc pallor from colour fundus photographs using previously validated software, *PallorMetrics*.^27^ Briefly, a set of deep learning-based convolutional neural networks segmented the optic disc to the inner edge of the border tissue and calculated pallor based on the reflectance of red and green light in the neuroretinal rim relative to a control region. Visible retinal vasculature was segmented and subtracted from measurement and control regions. Measurements of pallor were then obtained for seven zones, in concordance with zones used for peripapillary OCT scans and aligned with the fovea, which was automatically detected by the software. Key stages in *PallorMetrics* for a single image are presented in figure 1.

When *PallorMetrics* failed at segmentation, we manually annotated features by fitting a deformable ellipse to the inner edge of the optic disc border tissue and marking the fovea with a single mouse click; if the fovea was not visible, we estimated its location based on its position relative to the optic disc and the vessel arc of the central arcades (procedure detailed in Gibbon *et al*^27^). Annotations were performed by the author, SG, who was masked to stroke type.

### Sample derivation

Of the 186 patients recruited, 354 fundus photographs were available. Of the 354 images presented to the software, 73 had segmentation errors and were subsequently annotated manually. Via visual inspection of all processed images, eight were rejected for one or more rotation errors (two images), overexposure (two images) and high myope (five images). The final sample contained 346 images from 184 patients. For comparison, the final sample in our previous work using the same dataset was 166.^15^

### Statistical analysis

We used logistic regression to assess the relationship between disc pallor and stroke type and ordinal logistic regression to assess the relationship between pallor and total SVD score. In accordance with previous work,^13 15^ we controlled for age, sex, hypertension and diabetes; however, we included information on hyperlipidaemia, history of transient ischaemic attack, history of stroke and smoking status to table 1 for completeness. We also controlled for disc area. Correcting for multiple comparisons was not applied in this study because the primary aim was exploratory, intended to identify potential associations that could guide future research. The right eye was consistently paler than the left (table 1); therefore, the left and right eyes were assessed separately. Pallor, age and disc area were standardised before analysis. Differences in demographics and risk factors between the groups were assessed using t-tests (where one variable was continuous) or χ^2^ (where both variables were dichotomous). Missing data points were removed at the time of analysis. All statistical analysis was carried out in R (V.4.2.1; www. R-project.org). We used the ‘polyr’ package for ordinal logistic regression.

**Table 1:** Demographics and study variables by stroke subtype and eye

Variable	Stroke type				P value
	Cortical		Lacunar		
N (participants)	92		92		
N (eyes)	174		172		
Age	70.0 (11.2)		66.1 (11.5)		0.017
Sex (female)	48 (27.6%)		68 (39.5%)		0.117
Small vessel disease score					
0	46 (50%)		35 (38.0%)		
1	20 (21.7%)		16 (17.4%)		
2	16 (17.4%)		20 (21.7%)		
3	8 (8.7%)		13 (14.1%)		
4	2 (2.2%)		5 (5.4%)		
Missing	0 (0.0%)		3 (3.3%)		
Risk factors					
Hypertension (years)	112 (64.4%)		100 (58.1%)		0.543
Hyperlipidaemia (years)	65 (37.4%)		61 (35.5%)		1.000
Missing	0 (0.0%)		4 (2.3%)		
Diabetes (years)	20 (11.5%)		34 (19.8%)		0.225
Smoking (ever)	101 (58.0%)		97 (56.4%)		1.000
Missing	1 (0.6%)		1 (0.6%)		
Transient ischaemic attacj history (years)	17 (9.8%)		25 (14.5%)		0.273
Stroke history (years)	17 (9.8%)		17 (9.8%)		
Optic disc pallor					
Eye	Left	Right	Left	Right	
N (eyes)	86	88	88	84	
Global	1.16 (0.12)	1.16 (0.11)	1.16 (0.09)	1.21 (0.12)	
Temporal	1.21 (0.14)	1.23 (0.14)	1.22 (0.13)	1.28 (0.15)	
Temporal-inferior	1.15 (0.13)	1.16 (0.12)	1.15 (0.11)	1.21 (0.12)	
Nasal-inferior	1.07 (0.12)	1.06 (0.10)	1.08 (0.09)	1.10 (0.11)	
Nasal	1.14 (0.13)	1.14 (0.10)	1.15 (0.09)	1.17 (0.13)	
Nasal-superior	1.12 (0.12)	1.12 (0.10)	1.13 (0.09)	1.17 (0.11)	
Temporal-superior	1.12 (0.13)	1.12 (0.11)	1.13 (0.10)	1.17 (0.13)	
Papillomacular bundle	1.23 (0.15)	1.25 (0.14)	1.23 (0.13)	1.30 (0.15)	
Nasal-temporal ratio	0.947 (0.07)	0.931 (0.06)	0.951 (0.09)	0.915 (0.07)	
Retinal covariates					
Disc area	106 000 (29 000)	103 000 (26 700)	103 000 (23 900)	105 000 (21 600)	

Notes: Values are N (%) or mean (SD).

## Results

Optic disc pallor was measured in 184 patients (346 images). The mean age was 68.6 years (SD=11.5) and the majority sex was male (66.8%). There were 92 patients with cortical stroke and 92 with lacunar stroke. Individuals who experienced lacunar stroke were younger than those with cortical stroke (mean age 66.1 compared with 70, p=0.017). There was no evidence of significant differences in male/female split, hypertension, cholesterol, diabetes, history of stroke, history of transient ischaemic attack or smoking status between stroke types. Demographic and study variables are summarised in table 1.

### Associations between disc pallor and stroke type

**Figure 2:**
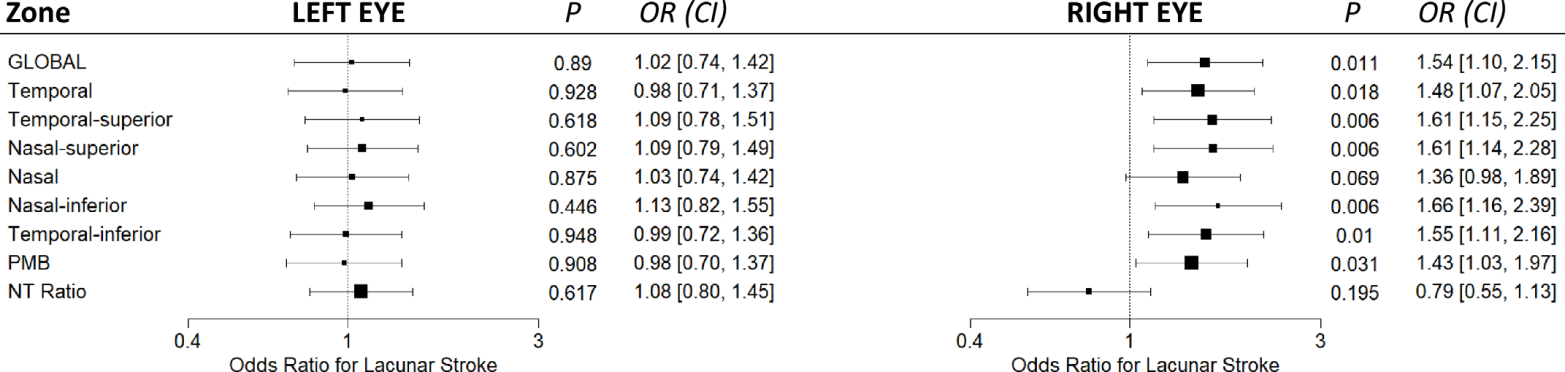
Forest plot showing the associations between disc pallor and stroke type. Each eye is modelled separately. All models are adjusted for age, sex, disc area, hypertension diagnosis and diabetes. Continuous variables were standardised prior to analysis. PMB, papillomacular bundle; NT, nasal-temporal.

In the right eye, independent of covariates, increased optic disc pallor was significantly associated with lacunar stroke globally (OR per SD increase=1.54, CI: 1.12 to 2.19, p=0.011) and across all zones (figure 2). The left eye did not reach statistical significance; however, the direction was mostly the same, potentially indicating a small effect.

### Associations between disc pallor and total SVD score

Ordinal logistic models of the entire sample (lacunar and cortical) revealed significant associations between pallor and total SVD score in the temporal superior (standardised β=0.36, SE=0.15, p=0.020) and nasal-inferior zones (standardised β=0.44, SE=0.15, p=0.004) in the right eye. The direction of β was the same in both eyes (except for the NT ratio in the right eye and nasal superior zone in the left eye), indicating that a statistical effect may have emerged given more data. Results are summarised in table 2.

**Table 2:** Ordinal logistic regression results for SVD score and optic disc pallor, by zone and eye

Zone	Coefficients			
	Left eye		Right eye	
	β (SE)	P value	β (SE)	P value
Global	0.07 (0.15)	0.646	0.26 (0.16)	0.101
Temporal	0.05 (0.16)	0.756	0.24 (0.16)	0.124
Temporal-superior	0.14 (0.15)	0.350	0.36 (0.15)	0.020*
Nasal-superior	−0.03 (0.15)	0.844	0.09 (0.16)	0.583
Nasal	0.06 (0.15)	0.700	0.21 (0.16)	0.188
Nasal-inferior	0.19 (0.15)	0.210	0.44 (0.15)	0.004**
Temporal-inferior	0.02 (0.15)	0.878	0.13 (0.16)	0.422
Papillomacular bundle	0.04 (0.16)	0.822	0.22 (0.16)	0.164
Nasal-temporal ratio	0.04 (0.15)	0.804	−0.12 (0.15)	0.412

All models were adjusted for age, sex, disc area, hypertension diagnosis and diabetes. Age, pallor and disc area were standardised prior to analysis.

*p<0.05 and **p<0.01.

## Discussion

We show here that the optic disc is slightly paler in patients with lacunar stroke compared with cortical stroke. This effect was independent of covariates and held despite the patients with lacunar stroke being around 4 years younger than patients with cortical stroke. Furthermore, we show that increasing paleness of the disc may be weakly associated with total SVD score. Both findings were stronger for the right eye; however, this may be due to the imaging protocol and known differences between the eyes. Taken together, our findings suggest that SVD may be associated with damage to the optic nerve.

A pale optic disc is symptomatic of optic atrophy, which refers to the permanent loss or damage to retinal ganglion cell axons along the anterior visual pathway, with pallor usually beginning to show around 4–6 weeks after axonal damage.^19^ The retinal imaging in the current study was carried out within 4 weeks of stroke occurrence; therefore, it is unlikely that the stroke itself caused the observed increase in paleness. More likely is that the observed increase in pallor is a long-term result of SVD, which has been associated with pRNFL thinning,^12^ and by association—disc pallor. This hypothesis is consistent with the current demonstration of associations between disc pallor and both lacunar stroke and SVD severity.

One possible mechanism by which SVD and resulting lacunar stroke could cause optic disc pallor/atrophy is through effects on the blood supply to the optic nerve or its pathways. For example, if a lacunar infarct occurred in any part of an optic pathway, it could lead to retrograde demyelination along the optic nerve, manifesting as pallor in the optic disc. However, this scenario is rare, as lacunar strokes do not usually affect the visual system directly.^34^ Optic disc pallor can reflect not only the loss of nerve fibres but also gliosis,^35^ axonal degeneration and other changes within the optic nerve head. It indicates chronic, longstanding optic nerve damage, which may be more aligned with the cumulative effects of chronic SVD.

Strengths of this study include the accuracy of stroke subtyping, which used both clinical and radiological features, the relatively large sample and the use of novel, validated software to quantify optic disc pallor in several zones.

This study has several limitations. First, associations were only statistically significant in the right eye and not the left. However, it is worth noting that the right eye was always captured first, using six flashes of light. This procedure may have had a subtle impact on image quality in the fellow eye, potentially due to patient fatigue or challenges in visualising the left optic disc. This claim remains to be tested and is beyond the scope of this study; empirically assessing image quality in fundus photographs is an area of much research and controversy.^36^ Notwithstanding, associations in the left eye showed a trend, which may have borne out in a larger sample. Furthermore, there are known differences in RNFL thickness between the eyes,^37^ which may have subtly affected the pallor measurement. Ocular dominance may also play a role; if patients are right-eye dominant, this could potentially lead to a thicker RNFL,^38^ which may result in greater variation in pallor measurements. This increased variation could potentially enhance the ability to detect differences or trends through statistical testing. Unfortunately, neither ocular dominance nor handedness (a proxy) was available for the current cohort.

Second, although patients were screened for glaucoma, they were not screened for other optic neuropathies, such as non-arteritic anterior ischaemic optic neuropathy (NAAION), which could affect disc pallor. However, NAAION is rare, affecting between 2.3 and 10.3 people per 100 000,^39^ making it unlikely that any patients in the study were affected. Even if one or two patients were affected, it is improbable that this would have significantly impacted the model estimates. Additionally, markers of optic disc dysfunction, such as colour vision deficiency and reduced visual acuity, which likely correlate with optic disc pallor, could have been useful covariates. Unfortunately, these variables were not collected.

Third, the study was cross-sectional in design, which precluded us from inferring a temporal association. In addition, most patients in the current study were white in ethnicity; therefore, further research is needed to determine if the results apply to other ethnicities. Finally, although we used MRI in deciding each stroke type and all patients had a definite stroke diagnosed by an expert panel, there may be some misclassification in stroke type. The classification used was a pragmatic system based on the most likely mechanism to explain the different stroke subtypes. We acknowledge that there will be a small group of people whose lacunar stroke may not have been caused by intrinsic SVD and also that there will be patients with coexistent small and large artery disease given the high prevalence of shared risk factors.

## Conclusion

We show here that the optic disc is slightly paler in lacunar compared with cortical stroke and that increasing disc pallor is weakly associated with increasing SVD severity. This may reflect RNFL loss or degeneration caused by vascular damage to the optic pathway. Our results strengthen the case for retinal fundus imaging as a convenient and promising method for exploring alterations in brain health linked to SVD.

## Data Availability

Data are available upon reasonable request.
